# Quarterly Meeting of the Rhode Island Medical Society

**Published:** 1879-01

**Authors:** 


### QUARTERLY MEETING OF THE RHODE ISLAND MEDICAL SOCIETY.

The regular quarterly meeting of the Rhode Island Medical Society was held in Providence, December 18, 1878, the president, Dr. E. T. Caswell, in the chair. The committee appointed at the last meeting to make collections for the benefit of the families of physicians at the South who died from yellow fever during the late epidemic, reported that they had collected and forwarded to the secretary of the Mississippi Medical Society the sum of one hundred and twenty dollars.

*Medical Witnesses.*—Dr. Turner, of Newport, made some remarks in regard to the compulsory attendance of physicians as witnesses in courts. He thought that some action should be taken to provide a law, if possible, by which courts should have discretion to allow a reasonable compensation, as done in some other States. In the present state of the law in Rhode Island it frequently works great hardship in compelling long attendance with no adequate pay.

Dr. Garvin, of Lonsdale, in the course of remarks upon the same subject, stated that on one occasion, being under summons to attend court at a certain hour, he found himself at that time in the midst of a case of labor, which of course he could not leave. On his arrival at court, half an hour late, he was informed that a writ had been issued for his arrest. But upon explanation of the circumstances he was allowed to escape further penalty upon payment of costs.

Upon motion of Dr. Turner, a committee consisting of Drs. Garvin, Kenyon and Dedick, was appointed to inquire what measures could be taken to procure legislation that would remedy this evil.

*Metric System.*—The report of the committee upon the adoption of the metric system was read by Dr. J. W. Mitchell, of Providence. Upon recommendation of this committee, a resolution was adopted that on and after January 1, 1880, the metric system should be used by the Fellows of this society in the writing of prescriptions.

*Medical Examiners.*—Dr. H. W. Williams, of Boston, was introduced, and gave an account of the recently established system of medical examiners in the State of Massachusetts.

He described the workings of the new law, and demonstrated fully its superiority over the old coroners' inquest.

*Catarrh.*—Dr. H. G. Miller, of Providence, made some remarks upon the treatment of naso-pharyngeal catarrh. He deprecated the use of saline and other liquid applications by means of the nasal douche, as liable to produce injury of the middle ear. He recommended that all remedial agents should be applied in the form of dry powder by insufflation.

*Dysmenorrhœa.*—Dr. Virgil O. Hardon, of Providence, read a paper upon Mechanical Dysmenorrhœa. He described the symptoms and pathology of this affection, and advocated the treatment by incision of the cervix uteri after the manner of Sims. He cited a number of cases from his own practice which had been successfully treated in this way. This paper was followed by an animated discussion. Most of the participants were of the opinion that in the majority of cases of mechanical dysmenorrhœa an operation is not necessary, but that relief may be obtained by dilatation of the cervix uteri or by the use of anodynes.

*Diphtheria.*—Dr. J. O. Whitney, of Pawtucket, read an elaborate essay upon diphtheria, in which he brought forward some novel ideas in regard to the ætiology and pathology of the disease. He maintained that there is but one pseudo-membranous disease, which has at different times borne a variety of names; such as diphtheria, croup, putrid sore throat, membranous laryngitis, cynanche maligna, etc. It is contagious or infectious in proportion to its visible putridity in individual cases, the contagious principle existing both in the breath of the sick and in the more solid discharges from the affected surfaces. It is primarily a local disease, the constitutional results depending upon absorption of the decomposed membrane. One attack gives no protection against a future attack.—*Boston Medical and Surgical Journal.*

